# High union rates following surgical treatment of proximal fifth metatarsal stress fractures

**DOI:** 10.1007/s00167-021-06490-2

**Published:** 2021-02-22

**Authors:** Julian J. Hollander, Quinten G. H. Rikken, Jari Dahmen, Sjoerd A. S. Stufkens, Gino M. M. J. Kerkhoffs

**Affiliations:** 1 Department of Orthopaedic Surgery, Amsterdam Movement Sciences, Amsterdam UMC, Location AMC, University of Amsterdam, Meibergdreef 9, 1105 AZ Amsterdam, The Netherlands; 2 Academic Center for Evidence Based Sports Medicine (ACES), Amsterdam UMC, Amsterdam, The Netherlands; 3 Amsterdam Collaboration for Health and Safety in Sports (ACHSS), International Olympic Committee (IOC) Research Center, Amsterdam UMC, Amsterdam, The Netherlands

**Keywords:** Fifth metatarsal, Stress fracture, Conservative, Surgery, Non-surgical

## Abstract

**Purpose:**

The primary purpose of this study was to determine the union rate and time for surgical- and non-surgical treatment of stress fractures of the proximal fifth metatarsal (MT5). The secondary purpose was to assess the rate of adverse bone healing events (delayed union, non-union, and refractures) as well as the return to sports time and rate.

**Methods:**

A literature search of the EMBASE (Ovid), MEDLINE (PubMed), CINAHL, Web of Science and Google Scholar databases until March 2020 was conducted. Methodological quality was assessed by two independent reviewers using the methodological index for non-randomized studies (MINORS) criteria. The primary outcomes were the union time and rate. Secondary outcomes included the delayed union rate, non-union rate, refracture rate, and return to sport time and rate. A simplified pooling technique was used to analyse the different outcomes (i.e. union rate, time to union, adverse bone healing rates, return to sport rate, and return to sport time) per treatment modality. Additionally, 95% confidence intervals were calculated for the union rate, adverse bone healing rates, and the return to sport rate.

**Results:**

The literature search resulted in 2753 articles, of which thirteen studies were included. A total of 393 fractures, with a pooled mean follow-up of 52.5 months, were assessed. Overall, the methodological quality of the included articles was low. The pooled bone union rate was 87% (95% CI 83–90%) and 56% (95% CI 41–70%) for surgically and non-surgically treated fractures, respectively. The pooled radiological union time was 13.1 weeks for surgical treatment and 20.9 weeks for non-surgical treatment. Surgical treatment resulted in a delayed union rate of 3% (95% CI 1–5%), non-union rate of 4% (95% CI 2–6%) and refracture rate of 7% (95% CI 4–10%). Non-surgical treatment resulted in a delayed union rate of 0% (95% CI 0–8%), a non-union rate of 33% (95% CI 20–47%) and a refracture rate of 12% (95% CI 5–24%), respectively. The return to sport rate (at any level) was 100% for both treatment modalities. Return to pre-injury level of sport time was 14.5 weeks (117 fractures) for surgical treatment and 9.9 weeks (6 fractures) for non-surgical treatment.

**Conclusion:**

Surgical treatment of stress fractures of the proximal fifth metatarsal results in a higher bone union rate and a shorter union time than non-surgical treatment. Additionally, surgical and non-surgical treatment both showed a high return to sport rate (at any level), albeit with limited clinical evidence for non-surgical treatment due to the underreporting of data.

**Level of evidence:**

Level IV, systematic review.

## Introduction

Stress fractures are partial or complete fractures that arise due to a repetitive load that is inferior to the stress required to break the bone within a single load [10]. A site of high incidence for stress fractures is the proximal fifth metatarsal (MT5), especially among (high-level) athletes [28]. Stress fractures of the proximal MT5 are problematic injuries as their inherent hypo-vascularity can lead to poor bone healing, which can result in a prolonged union time or even non-union [3, 30]. These complications can, in turn, affect the time to return to sports or work [8].

The treatment of proximal MT5 stress fractures can either be non-surgical or surgical [5, 9]. A preference for early surgical treatment of MT5 stress fractures exists in the literature as it seems to yield better bone healing outcomes and as well as a shorter bone union time in comparison to non-surgical treatment [3, 23]. This suggests that surgical treatment is the preferred treatment method for MT5 stress fractures. To date, however, there are no existing studies that have specifically pooled the union outcomes for MT5 stress fractures that have been previously reported in the literature. Additionally, no overview is available on the return to sports time and rate after both surgical and non-surgical treatment. Consensus for the optimal treatment of MT5 stress fractures is, therefore, limited [12, 17, 27]. The union rate is an important clinical indicator for successful fracture treatment, and the findings of this study may aid surgeons in optimally treating patients with a MT5 stress fracture. The primary purpose of this study is to determine the union rate after both surgical and non-surgical treatment. The hypothesis of the present study is that surgical treatment will lead to a higher pooled union rate compared to non-surgical treatment. The secondary purpose is to determine the union time, the rate of adverse bone healing events (i.e. delayed- and non-union rates and refracture rate), and the return to sport time and rate.

## Materials and methods

The study protocol was prospectively registered in an international prospective registry for systematic reviews, PROSPERO [4], with registration number: CRD42020178295. The preferred reporting items for systematic reviews and meta-analyses (PRISMA) statement was used as a guideline for this study [16].

### Search strategy

Studies from the first available record until March 2020 from EMBASE (Ovid), MEDLINE (PubMed), CINAHL, Web of Science and Google Scholar were searched. The full search strategy is available in the Appendix. Backwards citation chaining was applied to search for any additional eligible articles.

### Eligibility criteria and study selection

All clinical studies that investigated either surgical or non-surgical treatment of proximal MT5 stress fractures were included. Furthermore, studies written in English, French, German and Dutch were eligible for inclusion. There were no restrictions regarding patient age, demography or the date of publication. The exclusion criteria are shown in Table 1. If the results of acute and stress fractures were combined in an eligible study, or if individual data for stress fractures was not presented, the corresponding author was contacted by e-mail to ask for the provision of additional data. Authors were also contacted in situations where it was unclear whether stress fractures were included or not. If no response was received, two reminder e-mails were sent. If the corresponding authors remained unresponsive, the article was excluded. Two independent reviewers (J.H. and Q.R.) performed the title and abstract screening, as well as the full-text screening, using Rayyan [24]. In case of disagreement, an attempt was made to reach consensus. When no consensus was reached, the senior author (G.K.) was decisive.

**Table 1:** Exclusion criteria

Follow-up less than 3 months
Less than ten stress fractures reported
Reoperation as primary treatment following refracture or non-union
Review-, cadaver- and animal studies
Patient overlap

### Methodological quality

The methodological quality was evaluated by two independent reviewers (J.H. and Q.R.) using the Methodological Index for Non-Randomized Studies (MINORS) [29]. When no agreement was achieved after discussion, a third author (J.D.) was decisive.

### Data extraction

Data extraction was independently performed by two reviewers (J.H. and Q.R.) using a standardized extraction form specifically designed for the present study and that was piloted before use. Authors cross-verified extracted data before analysis. Stress fractures were defined as a fracture with clinical or radiological signs of a chronic stress reaction to the proximal MT5 and included as determined by the respective authors [6, 9, 25]. Additionally, Torg type two and three fractures were also included as stress fractures in this review [31].

Data on study characteristics, patient characteristics, bone healing outcomes, and sport outcomes were collected. Study characteristics included author, title, level of evidence, year of publication, treatments reported, and follow-up duration. Patient data included gender, age, body mass index (BMI), activity level and fracture location according to Lawrence and Botte [14]. The bone healing outcomes that were extracted included the number of unions, time to radiological union, number of delayed- and non-unions, and the number of refractures. Sport outcomes included the rate of return to any/pre-injury level of sports and return to any/pre-injury level of sports time. If return to sport was not specified for pre-injury level or any level, it was considered as return to sports at any level. Additionally, all patient-reported and clinical outcome measures were extracted.

### Statistical analysis

Descriptive variables were displayed as means with ranges for continuous variables, and absolute numbers and frequencies for categorical variables. Ranges of the reported pooled means and proportions include the lowest and highest mean values from the included studies. Time units were converted to either weeks or months, depending on the outcome variable. Due to the limited number of comparative studies, a formal meta-analysis was not carried out. Instead, a simplified pooling method was used, whereby pooled means and proportions were weighted by the number of fractures per study for each treatment modality (i.e. surgical or non-surgical treatment).

The primary outcome—namely, the union rate—was defined as the proportion of unions per treatment modality, excluding refractures. The union rate was also pooled for specific treatments within both treatment modalities (e.g. casting for non-surgical treatment). Additionally, a sub-analysis of the union rate for both treatment modalities per anatomical Lawrence and Botte zone was performed. For the secondary outcomes, the union time was pooled and weighted by the number of fractures per study. This was carried out for each treatment modality and their specific treatments. Furthermore, the rate of adverse bone healing events (i.e. the delayed union rate, non-union rate, and refracture rate) was calculated as the percentage of total fractures per treatment. To analyse the return to sport two different categories were used: return to sport at any level and return to sport at pre-injury level [1]. 95% confidence intervals (95% CI) were calculated within each pooled treatment modality and specific treatment group for the union rate, the rate of adverse bone healing events, and the return to sport rate using the Wilson score method (without continuity correction) [22]. All analyses were performed in STATA 15 (StataCorp LP, College Station, TX).

## Results

### Article selection

**Fig. 1:**
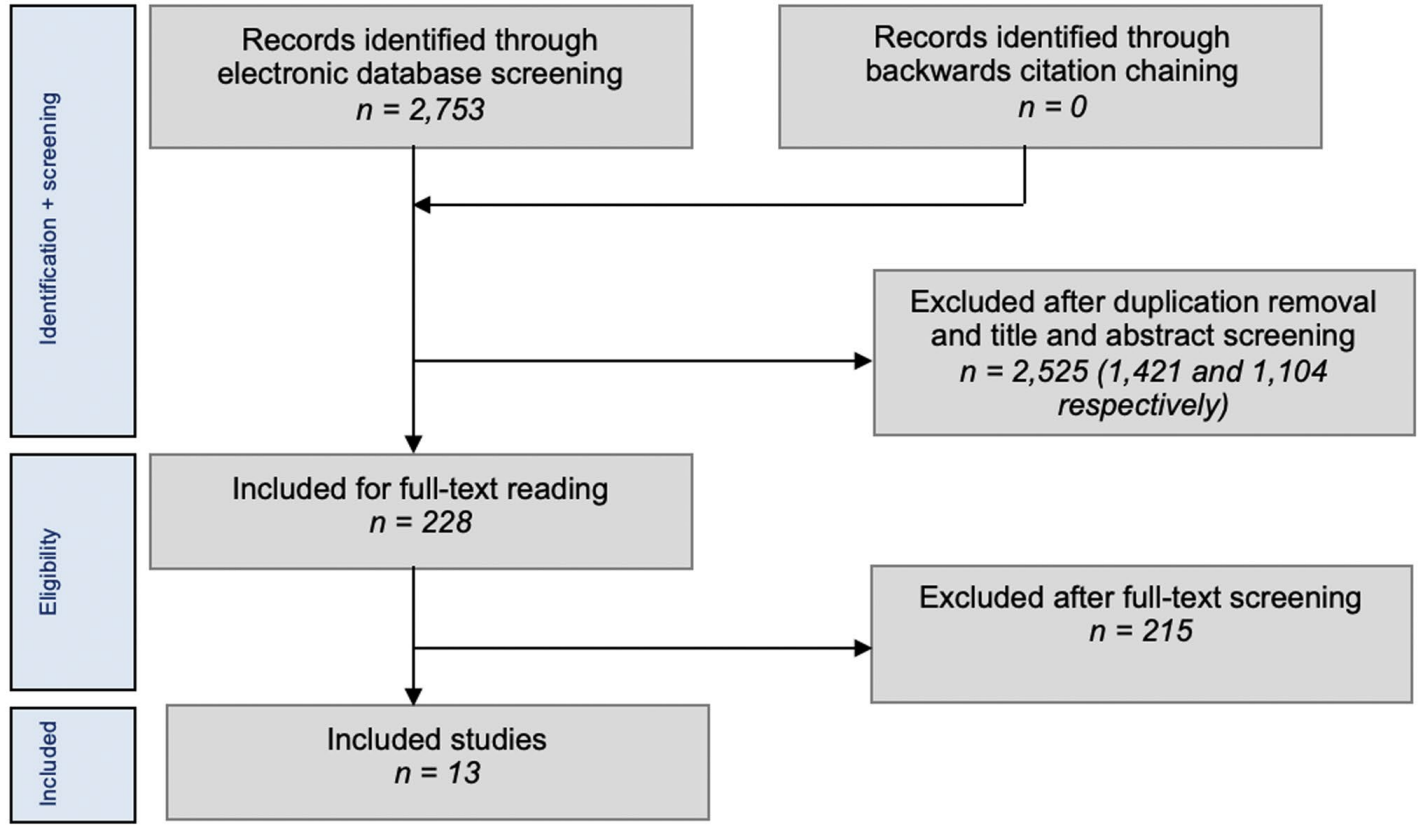
PRISMA flowchart of study selection

The literature search resulted in 2753 records, of which thirteen were included for final analysis after screening and contacting corresponding authors (Fig. 1). One prospective case series, nine retrospective case series and three retrospective comparative studies were included. A total of 393 stress fractures of the proximal MT5 were included in this review, with a mean age of 21.4 years (range: 19–28).

### Methodological quality

The authors reached consensus on the MINORS score for each study. Non-comparative studies scored an average of 7.5 (range 4–12) out of 16 points [2, 6, 9, 11, 13, 18, 19, 21, 25, 33]. Comparative studies were scored at an average of 12.3 (range 12–13) out of 24 points [5, 15, 26]. The individual MINORS scores are available in the Appendix.

## Clinical outcomes

### Surgical treatment

Twelve studies reported outcomes of surgical treatment for a total of 350 fractures [2, 5, 6, 9, 13, 15, 18, 19, 21, 25, 26, 33]. Four different surgical methods were reported, of which intramedullary screw (IMS) was most frequently used (60%). The other techniques were tension band wiring (25%), plantar plating (11%), and medullary curettage (0.1%). The study- and patient characteristics can be found in Table 2. The bone union rate for surgical treatment was 87% (95% CI 83–90%). Moreover, the pooled time to radiological union was 13.1 (range 7.5–42.6) weeks. The sub-analysis for the union rate of surgically treated zone 2 and 3 fractures—regardless of specific treatment—showed a union rate of 91% (95% CI 82–95%) and 91% (95% CI 82–96%), respectively (Table 4). A full overview of the secondary outcomes and sports outcomes per specific treatment is available in Tables 2 and 3.

**Table 2:** Pooled patient characteristics and bone union outcomes^†^

Treatment		Studies *n*	Fractures^∆^ *n*	Age years (range)	Follow-up months (range)	Union rate % (95%CI)	Radiological union time weeks (range)	Delayed union rate % (95%CI)	Non-union rate % (95%CI)	Refracture rate % (95%CI)
Surgery	Overall	12‡	350	20.8 (19–28)	44.2 (14.5–123.6)	87 (83–90)	13.1 (7.5–42.6) *n* = *305*	3 (1–5)	4 (2–6)	7 (4–10)
	IMS	8^#^	210	21.1 (19–28)	48.3 (14.5–123.6)	91 (87–94)	14.8 (7.5–42.6) *n* = *181*	4 (2–8)	2 (1–5)	3 (1–6)
	Tension band wiring	1 [15]	86	20.2	N/A	80 (71–87)	11.3 *n* = *86*	0 (0–4)	11 (6–19)	9 (5–17)
	Plantar plating	1 [33]	38	19.7	23.0	90 (76–96)	9.1 *n* = *38*	0 (0–9)	0 (0–9)	11 (4–24)
	Medullary curettage and bone grafting	1 [13]	2	28.0	16.0	100 (34–100)	N/A	0 (0–66)	0 (0–66)	0 (0–66)
Non-surgical	Overall	4 [5, 9, 11, 13]	43	26.2 (23–28)	108.9 (16–255.7)	56 (41–70)	20.9 *n* = *8*	0 (0–8)	33 (20–47)	12 (5–24)
	Cast	3 [5, 11, 13]	14	27.5 (24–28)	38.9 (16–144)	64 (39–84)	20.9 *n* = *8*	0 (0–22)	29 (12–55)	7 (1–31)
	Combined*	2 [11, 13]	23	26.2 (24.8–28)	151.5 (16–255.7)	56 (37–74)	N/A	0 (0–14)	39 (22–59)	4 (1–21)

*RTS* return to sport, *IMS* intramedullary screw, *N/A* not applicable

^†^All continuous outcomes are depicted as pooled means

*Patients were treated with a combination of non-surgical treatments consisting of elastic bandaging and weight-bearing (*n* = 13), and variations of weight-bearing (*n* = 8). ∆ The article by Ekstrand et al.[10] contains 14 surgically and 7 non-surgically treated patients which could not be further categorized into specific treatment groups. The total of the specific treatment groups is therefore not equal to the number of fractures in the overall analysis

^‡^Reference Number: [2, 5, 6, 9, 13, 15, 18, 19, 21, 25, 26, 33]

#Reference Number: [2, 5, 6, 18, 19, 21, 25, 26]

**Table 3:** Pooled sport outcomes^†^

Treatment		RTS rate at any level % (95%CI)	RTS time at any level weeks (range)	RTS rate at pre-injury level % (95%CI)	RTS time at pre-injury level weeks (range)
Surgery^#^	Overall [2, 5, 6, 9, 15, 18, 19, 21, 25, 26, 33]	100 (98–100) *n* = *231*	10.1 (6.3–15.2) *n* = *253*	99 (96–100) *n* = *171*	14.5 (8.5–22.2) *n* = *117*
	IMS [2, 5, 6, 18, 19, 21, 25, 26]	100 (97–100) *n* = *107*	9.6 (6.3–15.2) *n* = *201*	98 (89–100) *n* = *47*	10.6 (8.5–12) *n* = *65*
	Tension band wiring [15]	100 (96–100) *n* = *86*	N/A	100 (96–100) *n* = *86*	N/A
	Plantar plating [33]	97 (87–100) *n* = *38*	11.6 *n* = *38*	97 (87–100) *n* = *38*	22.2 *n* = *38*
Non-surgical	Overall [5, 9, 11]	100 (7*8*–100) *n* = *14*	19.3 (9.9–26.3) *n* = *14*	N/A	9.9 *n* = *6*
	Cast [5, 11]	100 (21–100) *n* = *1*	26.3 *n* = *8*	N/A	N/A
	Combined* [11]	100 (77–100) *n* = *13*	N/A	N/A	N/A

*RTS* return to sport, *N/A* not applicable

^†^All continuous outcomes are depicted as pooled means

*Patients were treated with variations of weight-bearing (*n* = 13)

^#^No sport outcomes for medullary curettage and bone grafting were reported

### Non-surgical treatment

Four studies—comprising a total of 43 fractures—reported outcomes for non-surgical treatment [5, 9, 11, 13]. Patients were either treated with casting (33%) or a combination of non-surgical treatments such as bandaging and weight-bearing (53%). The study and patient characteristics are depicted in Table 2. The pooled bone union rate for non-surgical treatment was 56% (95% CI 41–70%) and the radiological union time was 20.9 weeks. Furthermore, the sub-analysis of the union rate per anatomical zone for non-surgical treatment showed a union rate of 65% (95% CI 43–82%) for zone 2 and 88% (95% CI 53–98%) for zone 3 fractures (Table 4). An overview of the union time and the rate of adverse bone healing events for non-surgical treatment is available in Table 2. Lastly, the pooled sports outcomes are reported in Table 3.

**Table 4:** Bone union outcomes per anatomical zone†

Zone	Treatment		Studies	Fractures* n	Age years	Follow-up months	Union rate % (95%CI)	Delayed union rate % (95%CI)	Non-union rate % (95%CI)	Refracture rate % (95%CI)
Zone 2	Surgery	Overall	2 [9, 21]	74	19.8	40.9	91 (82–95)	1 (0–7)	1 (0–7)	7 (3–15)
		IMS	1 [21]	60	19	40.9	97 (89–99)	2 (0–9)	2 (0–9)	0 (0–6)
	Non-surgical	Overall	2 [9, 11]	20	24.2	247.7	65 (43–82)	0 (0–16)	10 (3–30)	25 (11–47)
		Cast	1 [11]	1	24	144	0 (0–79)	0 (0–79)	0 (0–80)	100 (21–100)
		Weight-bearing	1 [11]	13	24.8	255.7	85 (58–96)	0 (0–23)	8 (1–33)	8 (1–33)
Zone 3	Surgery	Overall	4 [5, 18, 25, 26]	69	22.8	65.3	91 (82–96)	0 (0–5)	3 (1–10)	6 (2–14)
		IMS	4 [5, 18, 25, 26]	69	22.8	65.3	91 (82–96)	0 (0–5)	3 (1–10)	6 (2–14)
	Non-surgical	Overall	1 [5]	8	27.6	40.0	88 (53–98)	0 (0–32)	13 (2–47)	0 (0–32)
		Cast	1 [5]	8	27.6	40.0	88 (53–98)	0 (0–32)	13 (2–47)	0 (0–32)

^†^All continuous outcomes are depicted as pooled means, no subdivision per specific treatment per modality was made. *The article by Ekstrand et al.[10] contains 14 surgically and 7 non-surgically treated patients which could not be further categorized into specific treatment groups. The total of the specific treatment groups is, therefore, not equal to the number of fractures in the overall analysis

## Discussion

The most important finding of this study is that surgical treatment of proximal MT5 stress fractures results in a short union time and a high union rate, with few adverse bone healing events (delayed union, non-union and refractures). The pooled union rate was found to be lower and the rate of adverse bone healing events higher for non-surgically treated patients. Additionally, surgically treated patients were found to return to sports at any level sooner but at a similar rate as non-surgically treated patients, albeit with limited clinical evidence for non-surgical treatment.

### Fracture healing

The present study shows that surgical treatment of proximal MT5 fractures results in lower rates of adverse bone healing events (i.e. delayed union, non-union and refracture rates) when compared to non-surgical treatment. A systematic review by Mallee et al. [17] concerning stress fractures in high-risk regions of the lower leg similarly found fewer adverse healing events and a shorter return to sport time after surgical treatment of proximal MT5 stress fractures. The disparity in union rates between surgically and non-surgically treated fractures may be due to the prolonged fracture healing that is observed in stress fractures. Proximal MT5 fractures tend to show poor healing due to the limited blood supply and high load during sports with repetitive (micro)trauma [3, 28, 30]. Non-surgical treatment may, therefore, be unable to adequately stabilize these types of fractures that are frequently seen in physically active patient populations [2, 6, 9, 15, 18, 19, 21, 25, 26, 33]. Surgical treatment may address this problem by providing an immediate stable fracture reduction which could allow for better callus formation and, therefore, superior healing [7]. As a result, patients are able to return to (full) weight-bearing and sporting activities at a faster rate. The trend towards increased surgical fixation for the management of these difficult fractures is clear in the literature. It must be stated, however, that the preference of surgical fixation over non-surgical treatment may lead to (publication) bias towards surgical treatment. Moreover, the results reported for surgically treated cases are often from (high-level) athletic patients, which may introduce selection bias [2, 6, 9, 15, 18, 19, 21, 25, 26, 33]. The available clinical results for non-surgical treatment are very limited in comparison with surgical interventions. Although surgery provides better union outcomes, non-surgical therapy can still offer satisfying union outcomes and should always be considered in fracture treatment and patients with increased risk for surgical complications [5, 11]. It must also be pointed out that no formal statistical comparison between treatment modalities was made in the present study due to methodological considerations. Further research on both treatment modalities—non-surgical treatment in particular—is, therefore, necessary to provide more valid evidence for the superior treatment of MT5 stress fractures.

When interpreting the rate of delayed unions and non-unions, it should be noted that varying definitions were used in the studies included in this systematic review, or that the definitions were not reported [7, 10, 12, 14, 16, 20, 22, 34]. A uniform definition is needed to enable improved analysis of the adverse bone healing events in further research. Additionally, varying imaging modalities were used to determine the time to union. This could affect the interpretation of this finding as the individual imaging modalities may have different sensitivities in the detection of bony union [2, 5, 11, 15, 18, 19, 21, 25, 26, 33]. However, radiographs were the most commonly used imaging modality in the included studies, thus limiting any possible bias.

### Surgical techniques

Although it was not the aim of the present study to compare the different surgical techniques, it can be noted that the most commonly used technique was IMS fixation. Which type of surgical treatment leads to superior union rates and patient-reported outcomes remains to be elucidated. The present study found comparable union rates for fixation using an IMS, tension band wiring, and plating. The pooled refracture rate of IMS was the lowest when compared to the other techniques. Evidence for tension band wiring and plating was, however, limited. Except for one patient that was treated surgically after painful non-union that resulted from his/her primary non-surgical treatment [5], it was reported that all patients received primary surgical treatment without preoperative non-surgical therapy. Caution is warranted when interpreting these results due to the low number of studies.

### Fracture location

The present study did not find a difference in union rates between Lawrence and Botte zone 2 and 3 fractures when treated surgically. Non-surgical treatment resulted in a lower union rate for zone 2 fractures compared to surgery. A previous systematic review on acute proximal MT5 fractures similarly found higher union rates for surgically treated patients [27]. These findings drive the hypothesis that Jones’ (i.e. zone 2) fractures may be grouped into acute, sub-acute, and stress fractures as they are subject to increased stress forces and could, therefore, benefit from surgical fixation. Physicians should carefully consider the involvement of a stress phenomenon in zone 2 fractures and should take this into account when considering the optimal treatment.

### Return to sports

In both the surgical and non-surgical treatment groups, almost all patients return to sport at any level. It was not possible to pool the return to sport rate at preinjury level for non-surgical treatment due to the underreporting of data. Surgical treatment was found to show a sooner return to sports at any level over non-surgical treatment, although data was limited. It must be acknowledged, however, that more research is needed to further investigate the return to sports after MT5 stress fracture treatment and what factors may contribute to a faster return to play. Lastly, it must be mentioned that preventative measures for MT5 stress fractures may spare patients the need for a surgical procedure that is accompanied by inherent risks [2, 33].

### Methodological considerations

The findings of the present study should be interpreted in the context of its design and limitations. The level of evidence of the included articles can be considered low, exemplified by the MINORS score. Except for one study, all included articles were of the retrospective design.

Limitations of this study include the small number of non-surgically treated patients and the fact that a formal statistical analysis was not able to be performed. This means that the present study did not make a direct statistical comparison between the outcomes of surgically and non-surgically treated patients. Caution is therefore warranted when interpreting the findings of this study as a variety of specific treatments were included with varying treatment indications. Additionally, the present study included articles with varying definitions of a stress fracture, which could have introduced a bias. However, a significant effort was made to contact authors in case it was not clear whether stress fractures were included or when additional data was needed.

### Clinical implications and future perspectives

Using the currently available evidence, surgical treatment results in a high union rate for proximal MT5 stress fractures and may be considered as the optimal treatment. The present study highlights the healing complications seen after non-surgical treatment as well as the scarcity of data on the result of non-surgical treatment. The findings of the present study can aid physicians in determining the best treatment strategy for patients with a proximal MT5 stress fracture. Additionally, these findings can help physicians in the shared decision-making process with patients, and in the expectation management for return to sports and possible union complications after such a fracture.

Future research should focus on reporting prospectively gathered clinical and union outcomes for surgical and non-surgical treatment modalities and should further investigate the factors associated with union complications and the failure of non-surgical treatment. The additional use of blood products such as bone marrow aspirate concentrate (BMAC) could enhance the fracture healing and return to sports and could be a promising adjuvant therapy [20, 26, 32].

## Conclusion

The union rate for surgically treated proximal MT5 stress fractures was found to be high with few adverse bone healing events (delayed union, non-union and refractures), whilst non-surgical treatment resulted in a lower union rate. The pooled union time was found to be longer in non-surgically treated patients. Additionally, surgical and non-surgical treatment both showed a high return to sport rate (at any level), albeit with limited clinical evidence for non-surgical treatment due to the underreporting of data.

## Abbreviations

MT5: Fifth metatarsal
PRISMA: The Preferred Reporting Items for Systematic Reviews and Meta-Analyses
MINORS: Methodological Index for Non-Randomized Studies
BMI: Body mass index
RTS: Return to sport
95% CI: 95% Confidence interval
IMS: Intramedullary screw
N/A: Not applicable
